# Selective Requirement for Maintenance of Synaptic Contacts onto Motoneurons by Target-Derived trkB Receptors

**DOI:** 10.1155/2016/2371893

**Published:** 2016-06-28

**Authors:** Xiya Zhu, Patricia J. Ward, Arthur W. English

**Affiliations:** Department of Cell Biology, Emory University School of Medicine, Atlanta, GA 30322, USA

## Abstract

Synaptic contacts onto motoneurons were studied in mice in which the gene for the trkB neurotrophin receptor was knocked out selectively in a subset of spinal motoneurons. The extent of contacts by structures immunoreactive for either of two different vesicular glutamate transporters (VGLUT1 and VGLUT2), the vesicular GABA transporter, or glutamic acid decarboxylase 67 (GAD67) with the somata of motoneurons, was studied in wild type and trkB knockout cells in tamoxifen treated male and female SLICK-trkB^−/−^ mice. Selective knockout of the trkB gene resulted in a marked reduction in contacts made by VGLUT2- and GAD67-immunoreactive structures in both sexes and a significant reduction in contacts containing only glycine in male mice. No reduction was found for glycinergic contacts in female mice or for VGLUT1 immunoreactive contacts in either sex. Signaling through postsynaptic trkB receptors is considered to be an essential part of a cellular mechanism for maintaining the contacts of some, but not all, synaptic contacts onto motoneurons.

## 1. Introduction

Changes in circuitry of the central nervous system (CNS) are an important consequence of injury to peripheral nerves. Among these CNS changes is the withdrawal of synaptic inputs from the cell bodies of axotomized motoneurons following peripheral injury [1–3]. In the mouse spinal cord, both excitatory and inhibitory synapses are withdrawn, beginning approximately 3–5 days following injury [2]. Over time, many of the withdrawn terminals are restored, regardless of whether axon regeneration is successful [1]. However, the withdrawal of terminals, originating from primary afferent neurons and expressing vesicular glutamate transporter 1 (VGLUT1), progresses over time, preterminal axons degenerate, and synapse loss is permanent [1, 4].

In a previous study, we described mice in which the gene for the neurotrophin, brain derived neurotrophic factor (BDNF), was knocked out in an inducible and cell-type specific manner [2, 5]. We showed that, by knocking out this gene in a subset of spinal motoneurons, we were able to mimic the extent of withdrawal of both excitatory (VGLUT1+) and inhibitory (glutamic acid decarboxylase 67, GAD67+) synaptic inputs observed after peripheral axotomy, but only contacts onto postsynaptic motoneurons lacking the BDNF gene (termed knockout (KO) cells) and not those in which the gene had not been manipulated (termed wild type (WT) cells) [2]. Transection of the sciatic nerve in these mice results in withdrawal of these contacts from the WT motoneurons but not in more synaptic withdrawal from KO cells. After treatment of injured mice with two weeks of moderate daily exercise, no significant loss of either type input was noted on WT cells; in KO cells these contacts were withdrawn. We interpreted these results to mean that motoneuron BDNF functions as a key part of a cellular mechanism to maintain VGLUT1+ and GAD67+ synaptic inputs onto motoneurons. The marked reduction in motoneuron BDNF expression beginning shortly after peripheral axotomy [6, 7] was deemed a likely source of the observed postinjury synaptic withdrawal. Such a neurotrophin-based interpretation is consistent with earlier speculations [8] and with the observation that prolonged exposure of the proximal stump of cut nerves to different neurotrophins resulted in a restoration of synaptic inputs onto the axotomized motoneurons [9] or a restoration of the amplitude of monosynaptic excitatory postsynaptic potentials (EPSPs) in them [10, 11].

The simplest role that BDNF could play in such a mechanism would be as a retrograde signal from motoneurons. By binding to trkB receptors on synaptic terminals, BDNF could help to promote synaptic maintenance. However, trkB expression is substantial in postsynaptic motoneurons [7, 12–14], so that trkB-mediated stabilization of synaptic terminals via other mechanisms cannot be ruled out. One goal of this study was to use mice in which the trkB receptor is knocked out selectively in motoneurons to investigate its role in promoting synaptic stability.

In the cerebellum, hippocampus, and cortex, BDNF-trkB signaling has been associated with the development [15–17] and maintenance [18, 19] of inhibitory synaptic inputs. Treatments of cut nerves with different neurotrophic factors resulted in effects on different populations of synaptic inputs onto motoneurons [9–11, 20]. A second goal of this study was to investigate the relative composition of different types of synaptic inputs onto motoneurons in motoneuron-specific trkB knockout mice. Two types of excitatory synapses and two types of inhibitory synapses were studied.

Finally, testosterone is known to be an important regulator of both BDNF and trkB expression in neurons [14, 21]. The role of motoneuron BDNF in maintaining synaptic inputs onto axotomized motoneurons with exercise is known to rely on androgen receptor signaling [22]. Estrogen has also been shown to regulate the development of BDNF mRNA and protein in the rat hippocampus [23]. The third goal of this project was to investigate sex differences regarding the role of motoneuron trkB receptors in synaptic maintenance.

We show here that selective and inducible knockout of the trkB gene from postsynaptic motoneurons resulted in significant withdrawal of synaptic inputs from intact motoneurons in adult mice. The withdrawal was proportionally greater for GABAergic inhibitory inputs than for excitatory inputs, and no evidence for withdrawal of VGLUT1+ inputs was obtained. A preliminary report of some of these findings has been made [24].

## 2. Material and Methods

### 2.1. Animals

All experimental procedures conformed to the Guidelines for the Use of Animals in Research of the Society for Neuroscience and were approved by the Institutional Animal Care and Use Committee of Emory University. All of the mice used in this study were adults (>2 months old) weighing 21–26 grams. Because mice made null for trkB at the time of conception do not survive [25], conditional gene knockout was performed. Mice with loxP sites flanking the coding region of the trkB gene (trkB^f/f^, a generous gift of Dr. Luis Parada) [26] were bred with mice expressing both tamoxifen-inducible Cre and Yellow Fluorescent Protein (YFP) under the control of the thy-1 promoter, a construct that directs expression to neurons. These latter mice are known as SLICK (single-neuron labeling with inducible Cre-mediated knockout) [27]. Several strains of SLICK mice have been developed. Only mice of the B strain were used in this study. In mice of this strain, YFP (and tamoxifen-inducible Cre recombinase) is expressed in a subset of motoneurons in the spinal cord [2, 5, 27]. It is not known why only a subset of neurons express the transgene. In one group of eight SLICK::trkB^f/f^ mice (four males and four females), Cre recombinase was activated by two bouts of three consecutive days of oral treatment with the synthetic estrogen, tamoxifen, separated by two weeks. This treatment regimen has been shown to result in neuron-specific expression of Cre [13, 24]. Once Cre is expressed, permanent elimination of the trkB gene occurs in motoneurons containing YFP. Motoneurons not expressing YFP (or Cre) are assumed to be trkB+. We have characterized the trkB expression in motoneurons in these mice elsewhere [28]. Mice were used in experiments between six and 10 weeks after the end of the second round of tamoxifen treatments. The withdrawal of VGLUT1+ synapses from motoneurons after peripheral axotomy is progressive [29] but is virtually complete by six weeks after injury [1]. We also have shown that the proportion of the afferent axons forming synapses onto motoneurons that express YFP and also are assumed to be trkB− is negligible [2]. A second group of four male SLICK::trkB^f/f^ mice was not treated with tamoxifen and acted as a control. Numbers of mice in each group were chosen based on an* a priori* power sample size estimate, using a power of 0.8, *α* = 0.05, and the interanimal variability observed in our previous studies [2, 22].

### 2.2. Motoneuron Labeling

In all mice, the cell bodies of presumed motoneurons were identified either through retrograde labeling (two female mice) or by immunoreactivity to NeuN (eight male and two female mice). NeuN is a neuronal nuclear antigen present in the cytosol of all neurons [1]. We studied large neurons in lamina IX of the ventral horn of the L3–5 segments of the spinal cord that were immunoreactive for NeuN, presuming that they were motoneurons. Results using the two approaches were identical. In retrograde labeling experiments, the gastrocnemius and soleus muscles of isoflurane anesthetized mice were injected with 1 *μ*L each of the beta subunit of cholera toxin B conjugated to Alexa Fluor 555 (1 mg/1 mL) using a Hamilton syringe equipped with a 35 G injection needle. Injections were performed bilaterally. Three days after muscle injections, the mice were euthanized with a lethal dose of pentobarbital (150 mg/kg) and perfused with saline and periodic acid-lysine-paraformaldehyde fixative [30]. Spinal cord segments L3 through L5 were harvested and cryoprotected in 20% sucrose overnight. The spinal cords were sectioned transversely into 20 *μ*m thick sections using a cryostat and mounted on Superfrost Plus slides.

### 2.3. Immunohistochemistry

To visualize the synaptic terminals onto motoneurons, we reacted separate sets of sections with antibodies to different synapse-specific antigens (Table 1). Glutamate decarboxylase 67 (GAD67) is an enzyme in the synthetic pathway for the inhibitory neurotransmitter, gamma-aminobutyric acid (GABA). The vesicular GABA transporter (VGAT) functions in the loading of synaptic vesicles containing both GABA and the inhibitory amino acid transmitter, glycine [31]. All GAD67+ terminals also contain VGAT. Terminals that contain VGAT but not GAD67 contain only glycine [32]. There are three vesicular glutamate transporters which function in the loading of synaptic vesicles containing the excitatory transmitter, glutamate. The overwhelming majority of terminals in the ventral horn of the spinal cord containing the VGLUT1 isoform are from primary afferent neurons [33, 34]. Synaptic terminals containing vesicular glutamate transporter 2 (VGLUT2) are excitatory synaptic inputs from interneurons [35].

Sections were first incubated on slides for one hour at room temperature in buffer containing 0.1 M phosphate-buffered saline (PBS) with 0.4% Triton X (PBS-T) and 10% normal goat serum (NGS). Following this preincubation, the tissues on separate slides were incubated in different primary antibodies overnight in a humid chamber at 4°C. The GAD67 and VGAT antibody binding was followed by incubation with a goat anti-mouse secondary antibody conjugated to Alexa Fluor 647 (1 : 200). The binding of VGLUT1 or VGLUT2 was detected by goat anti-rabbit secondary antibody conjugated to Alexa Fluor 647 (1 : 200). Retrograde labeling of motoneurons was enhanced with rabbit anti-cholera toxin B (1 : 200) followed by a goat anti-rabbit secondary antibody conjugated to Alexa Fluor 546 (1 : 200). Motoneurons without retrograde labeling were reacted with either mouse or rabbit anti-NeuN (1 : 200), followed by goat anti-mouse or goat anti-rabbit secondary antibody conjugated to Alexa Fluor 546. Washes were performed with 0.1 M PBS. All slides were cover slipped with Entellan.

### 2.4. Image Analysis

**Figure 1:**
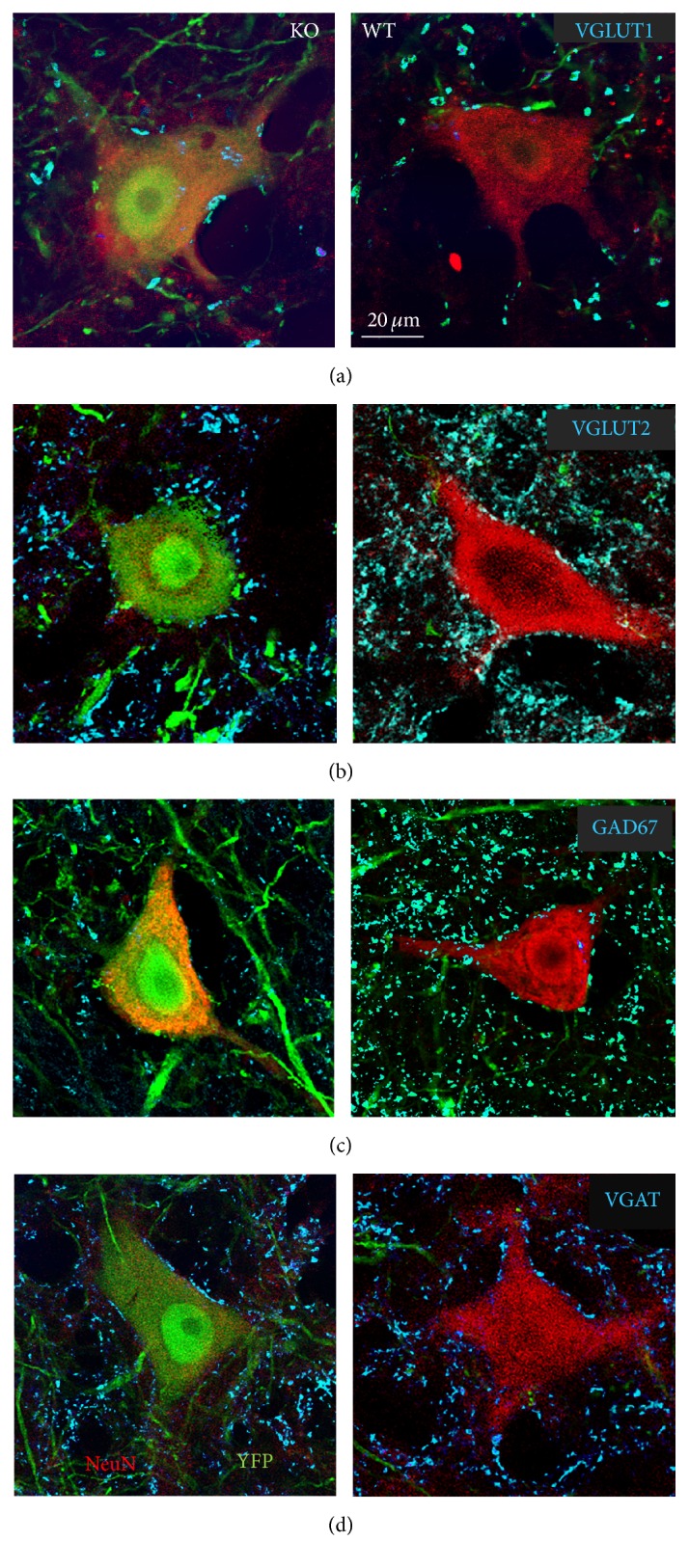
Immunoreactivity to different synapse-associated antigens surrounding the somata and proximal-most dendrites of large neurons in lamina IX of the L3–5 segments of the spinal cord of SLICK::trkB mice that had been treated with tamoxifen to knock out the trkB gene is shown. Cells expressing YPF (left column) are presumed to be null for the trkB gene and cells in the same histological sections that are YFP− are presumed to have normal expression of this gene. NeuN immunoreactivity (red) is shown to identify these cells as neurons. Because of their large size and laminar location they were considered motoneurons. Synaptic structures immediately adjacent to the perimeters of these cells (cyan) represent contacts made by structures immunoreactive to VGLUT1 (a), VGLUT2 (b), GAD67 (c), or VGAT (d). All cells are shown at the same magnification.

Images of single 1 *μ*m optical sections of cryostat sections were obtained using a Zeiss LSM510 confocal microscope at a magnification of 63x. Optical sections of presumed motoneurons were selected for study only if the location of the nucleus could be clearly visualized by the lack of immunoreactivity to either cholera toxin B or NeuN. Cell bodies of trkB knockout (KO) motoneurons innervating hind limb muscles were identified by the presence of both YFP (green) and either NeuN or cholera toxin B (red). In tamoxifen treated mice, these cells were assumed to be trkB null (KO). Cell bodies of motoneurons that did not contain YFP but were adjacent to YFP+ cells on the same sections and were immunoreactive for either NeuN or cholera toxin B were assumed not to express Cre recombinase and were considered wild type (WT). Images of 10 KO motoneuron cell bodies and 10 WT cells were obtained from each side of the spinal cord in each mouse. Immunoreactivity to GAD67, VGAT, VGLUT1, or VGLUT2 was visualized with far red (647 nm) optics. Representative images of trkB-KO (YFP+) and WT (YFP−) neurons from sections reacted with antibodies to the different synapse-associated antigens are shown in Figure 1. In the control group (SLICK::trkB^f/f^ mice not treated with tamoxifen), 20 YFP+ cells reacted with antibodies to each of the different synapse-associated proteins were selected from each side of the spinal cord for study. It was assumed that trkB expression in these cells was not affected.

**Figure 2:**
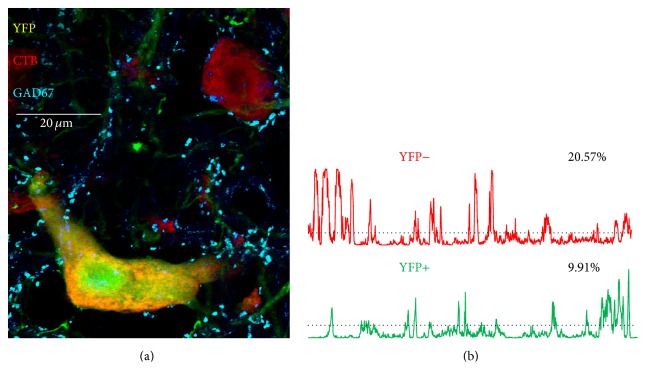
(a) Two motoneurons in a single optical section in lamina IX of the ventral horn of the lumbar spinal cord of a tamoxifen treated SLICK::trkB^f/f^ mouse are shown. Both neurons are marked by the presence of a red fluorescent retrograde tracer, cholera toxin B-Alexa Fluor 546, that had been injected into the gastrocnemius muscles three days prior to tissue harvesting, identifying these cells as motoneurons. One of the cells also expresses yellow fluorescent protein (YFP) and was assumed to be null for the gene for trkB. Cyan structures are immunoreactive for glutamic acid decarboxylase 67 (GAD67). (b) Profile plots are shown for GAD67 immunoreactivity along the perimeters of these cells. In each plot, the average fluorescence intensity + one standard deviation is shown as a threshold by the horizontal dashed line. Fluorescence intensity values greater than this threshold are assumed to represent contacts between the perimeter of the cell and structures immunoreactive for GAD67. The numbers next to each plot indicate the percent of the perimeter of the cell in such contact.

From the selected images of presumed motoneurons, the extent of contacts made by different types of synaptic inputs was measured using the computer program FIJI. A region of interest (ROI) was created to select the perimeter of each labeled motoneuron soma and very most proximal dendrites in the red channel of the RGB images, using the thresholding function of the software. The ROIs included only proximal-most dendrites, extending no longer than 25 *μ*m from the center of the cell somata, as the majority of the synapse withdrawal observed immediately following peripheral axotomy occurs in this region [1]. At more prolonged survival times, more subtle changes in VGLUT1+ synaptic contacts occur at more distal sites, as preterminal axon branches withdraw [4]. These changes were not investigated in this study. A plot profile (Figure 2(b), red and green lines), indicating the intensity of immunofluorescence for synapse-associated proteins beneath this outline (±1 micrometer width) of the boundary of the cell studied, was then generated from the blue channel of the RGB image, the component of the RGB image obtained using far red optics. Mean fluorescence intensity along this perimeter was determined and an intensity threshold was set at this mean plus one standard deviation about that mean [2, 36] (horizontal dashed lines in Figure 2(b)). All profile plot values greater than this threshold were assumed to indicate contact of the motoneuron soma by structures immunoreactive for one of the synapse-specific proteins. The proportion of the entire cell perimeter so contacted, that is, the proportion of the cell perimeter with fluorescence intensity values greater than threshold, was found for each cell and expressed as percent synaptic coverage.

### 2.5. Statistics

Two types of statistical analyses were performed to evaluate the effect of knocking out the trkB gene on the extent of contacts made by structures immunoreactive to synapse-associated proteins onto presumed motoneurons. First, for each of the four synaptic proteins studied, we pooled the measurements made from the neurons in all of the mice in the different groups studied: all cells in the control group and WT and KO cells, separately, in male and female, tamoxifen treated SLICK::trkB^f/f^ mice (four mice × 20 cells per mouse, *N* = 80 cells for each synapse type in each group). In each group, frequency distributions of synaptic coverage values were constructed. The distributions of coverages in the different groups were compared in pairs using the Mann-Whitney *U* test. This nonparametric method results in a probability that differences in the two distributions compared occur by chance only. Probabilities of <0.05 were considered significant.

Second, percent synaptic coverage in WT and KO cells in male and female tamoxifen treated SLICK::trkB^f/f^ mice and in control SLICK::trkB^f/f^ mice not treated with tamoxifen was subjected to a one way analysis of variance (ANOVA). Data from each of the four synaptic proteins studied were compared separately. If the omnibus test of the ANOVA was significant, then* post hoc* paired testing (Tukey's Honest Significant Differences, HSD) between groups was conducted. Probabilities of <0.05 were considered significant.

## 3. Results

### 3.1. VGLUT1+ Synapses in trkB-KO Mice

We determined the proportion of the perimeters of cells that were in contact with structures immunoreactive for VGLUT1 in presumed motoneurons of male and female SLICK::trkB^f/f^ mice that had been treated with tamoxifen. Data from cells presumed to contain no trkB (KO cells, YFP+) were compared to data from YFP− cells, presumed to be WT. Comparisons were made also to data from YFP+ neurons in control mice, SLICK::trkB^f/f^ mice that were not tamoxifen treated.

**Figure 3:**
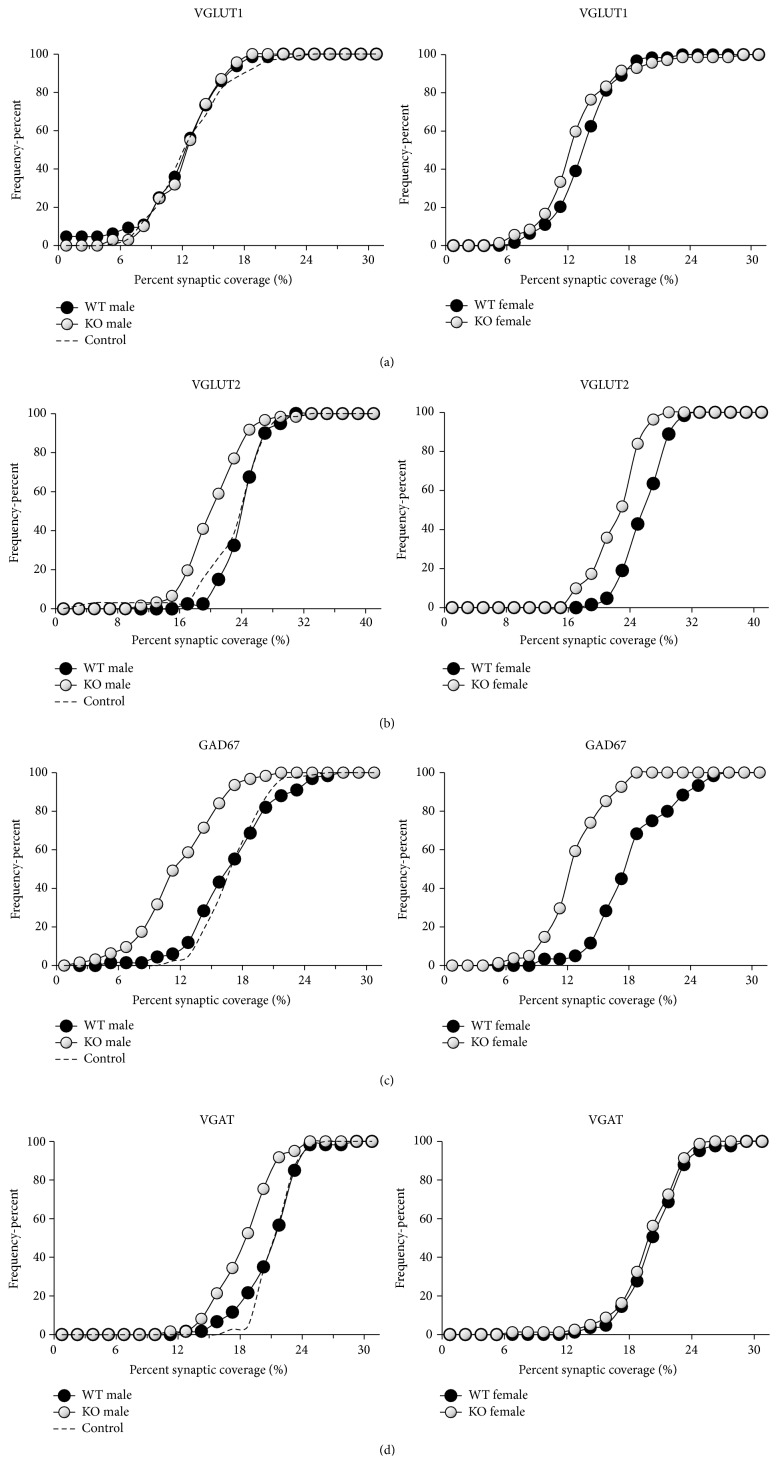
Cumulative frequency plots of percent coverage by structures immunoreactive to different synapse-associated proteins onto the soma and proximal-most dendrites of labeled motoneurons in tamoxifen treated and untreated SLICK::trkB^f/f^ male (left) and tamoxifen treated female (right) mice. WT (black symbols) refers to wildtype motoneurons in tamoxifen treated mice, without YFP, that still retain trkB receptors. KO (white symbols) refers to knockout motoneurons labeled with YFP that do not have trkB receptors. Control (dashed line) refers to data from male SLICK::trkB^f/f^ mice not treated with tamoxifen. Data from all the mice in each group were pooled. (a) VGLUT1. (b) VGLUT2. (c) GAD67. (d) VGAT.

We pooled data from all of the mice in each group and tested whether the frequency distributions of coverages by VGLUT1 immunoreactive contacts were significantly different between pairs of groups using the nonparametric Mann-Whitney *U* test. These distributions for VGLUT1+ contacts are shown in Figure 3(a). In male mice, no significant differences were found between the distributions of coverages in WT and KO cells, or between either of these and cells from control mice (Figure 3(a), left). In females, a slight but significant (*U* test, *p* < 0.04) shift to the left in the distributions of synaptic coverages onto KO cells was found relative to that of WT cells (Figure 3(a), right). This difference is due to slightly larger synaptic coverage in the WT cells of females (*U* test, WT male versus female, *p* < 0.05), as the distributions of coverages in the KO cells of both sexes were not significantly different (*U* test, n.s.).

**Figure 4:**
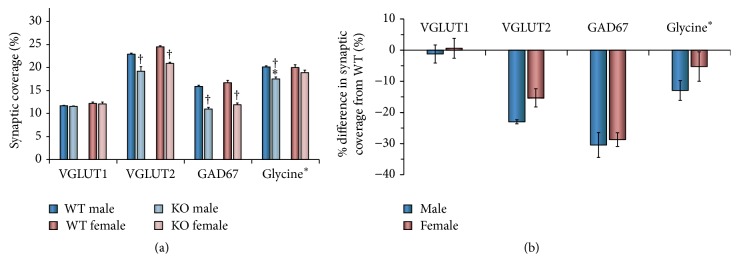
(a) The mean percent coverage (±SEM) by contacts immunoreactive to VGLUT1, VGLUT2, GAD67, or glycine in male and female tamoxifen treated SLICK::trkB^f/f^ mice. These values were determined for each synapse-associated protein by averaging data from individual mice in the different groups. The significance of differences between groups was then evaluated with ANOVA. The estimated percent coverage by contacts containing glycine equals the percent coverage by VGAT+ contacts minus percent coverage by GAD67. WT refers to wildtype motoneurons without YFP that still retain trkB receptors. KO refers to knockout motoneurons labeled with YFP that do not have trkB receptors. ^†^ *p* < 0.05 versus WT and ^*∗*^ *p* < 0.05 versus female. (b) The same data are expressed as mean (±95% confidence limits) percent differences between WT cells and KO cells.

In addition, we evaluated the significance of differences in mean synaptic coverage between groups using ANOVA. Mean (±SEM) percent synaptic coverages from WT and KO cells in treated and control animals are shown in Figure 4(a). The same data are expressed as percent change in synaptic coverage in Figure 4(b). No significant difference in percent VGLUT1 coverage was found between WT male, WT female, KO male, KO female, and controls (*F*_4,15_ = 1.76, n.s.).

### 3.2. VGLUT2+ Synapses in trkB-KO Mice

Data obtained from sections of spinal cords reacted with antibodies to VGLUT2, identifying glutamatergic synaptic inputs originating from interneurons, are displayed as pooled frequency histograms in Figure 3(b) and as mean synaptic coverage in Figure 4. In both males and females, the frequency histograms for VGLUT2 coverage from WT cells are not significantly different from those in control mice (*U* test, n.s.). In both sexes, the distributions of coverages onto KO cells are shifted significantly to the left of that for WT cells and all cells from control mice (Figure 3(b)) (*U* test, *p* < 0.01).

Mean coverage by VGLUT2 immunoreactive contacts differs significantly between the studied groups (*F*_4,15_ = 12.83, *p* < 0.01). Average percent synaptic coverage in WT cells of both sexes and in control mice did not differ significantly (HSD, n.s.). Mean coverage in KO cells is significantly (HSD, *p* < 0.01) smaller than found on WT cells, in both males and females (Figure 4(a)). The significant reduction in mean percent coverage by VGLUT2+ inputs in KO motoneurons is not significantly different between males and females (Figure 4(b)).

### 3.3. GAD67+ Synapses in trkB-KO Mice

Data obtained from sections of spinal cords reacted with antibodies to GAD67, associated with inhibitory GABAergic synaptic inputs originating from interneurons, are displayed as pooled frequency histograms in Figure 3(c) and as mean synaptic coverage in Figure 4. In males and females, no significant differences in the distribution of coverages by GAD67 immunoreactive contacts were found between WT cells of either sex and control motoneurons (*U* test, n.s.). In both sexes, a significant (*U* test, *p* < 0.01) shift to the left in the distribution of coverage by GAD67+ contacts for KO cells was found, relative to that for WT cells or cells from control mice (Figure 3(c)).

Mean coverage by GAD67 immunoreactive contacts differs significantly between the studied groups (*F*_4,15_ = 18.93, *p* < 0.01). Average percent synaptic coverage by GAD67+ contacts in WT cells of both sexes and in control mice did not differ significantly (HSD, n.s.). Mean coverage in KO cells is significantly (HSD, *p* < 0.01) smaller than found on WT cells, in both males and females (Figures 4(a) and 4(b)). No sex difference in GAD67 synaptic coverage was found for KO motoneurons (HSD, n.s.) (Figure 4).

### 3.4. Glycine+ Synapses in trkB-KO Mice

Data obtained from sections of spinal cords reacted with antibodies to VGAT, identifying inhibitory GABAergic and glycinergic contacts originating from interneurons, are displayed as pooled frequency histograms in Figure 3(d). For each mouse, we estimated the percent coverage by synapses containing glycine by subtracting the mean percent synaptic coverage for GAD67 from that of VGAT. The average of these differences (±SEM) is shown in Figure 4.

In both males and females, the distribution of coverages by VGAT immunoreactive contacts does not differ between WT cells and motoneurons from control mice (*U* test, n.s.). In both sexes, the distribution of coverages in KO cells is shifted significantly (*U* test, *p* < 0.01) to the left of that for WT cells in both males and females, suggesting a decrease in coverage by structures containing VGAT in cells made null for trkB. In male mice, the distribution of coverages by VGAT+ contacts in KO cells is shifted significantly (*U* test, *p* < 0.01) to the left of that for females, suggesting greater reduction in response to loss of trkB than found in females.

Mean estimated coverage by glycine-containing contacts differs significantly between the studied groups (*F*_4,15_ = 3.50, *p* < 0.03). Average estimated percent synaptic coverage by glycinergic contacts in WT cells of both sexes and in control mice did not differ significantly (HSD, n.s.). Mean estimated coverage in KO cells is significantly (HSD, *p* < 0.01) smaller than found on WT cells, only in males (Figures 4(a) and 4(b)). No significant effect of knocking out the trkB gene was noted for glycine-containing synaptic contacts in females (Figure 4).

## 4. Discussion

After peripheral nerve injury, connectivity between circuits in the central nervous system (CNS) and the musculoskeletal system are lost and changes within this circuitry are found. We have hypothesized [22] that these CNS changes could contribute to the poor recovery from peripheral nerve injuries observed clinically. One of these CNS changes is the withdrawal of synaptic terminals from the somata of the motoneurons [1, 3]. This withdrawal has been proposed to be due to a lack of neurotrophic support from the injured motoneurons [8], and this notion has been supported in previous publications from our lab [2] and others [9, 10, 20]. We have argued that BDNF secreted from motoneuron somata and proximal dendrites is a part of a cellular mechanism that maintains synaptic terminals onto motoneurons by binding to its receptor trkB on presynaptic terminals [2]. However, trkB receptors are also located on the postsynaptic motoneurons, and how signaling through these receptors may be involved in maintaining synapses on motoneurons is not clear.

The main finding of this study is that when trkB receptors are eliminated selectively from postsynaptic motoneurons, a significant withdrawal of synaptic terminals is found in otherwise intact mice. Contacts immunoreactive for VGLUT2 and GAD67, which originate from neurons within the CNS, with the somata of motoneurons lacking the trkB receptors were reduced relative to those found on trkB+ motoneurons in the same mice. The reduction in GAD67+ contacts is consistent with previous literature showing that BDNF-trkB signaling is associated with the specific development [15–17] and maintenance [18, 19] of inhibitory synaptic inputs in certain regions of the brain. The finding that trkB elimination in postsynaptic motoneurons results in a withdrawal of VGLUT2+ inputs is novel. We interpret these findings to mean that postsynaptic motoneuron trkB signaling plays a prominent role in the maintenance of excitatory VGLUT2+ as well as inhibitory GABAergic synapses. The functional consequences of these anatomical observations of selective withdrawal of synaptic inputs remain for future studies.

The binding of BDNF to trkB receptors on motoneurons can signal changes in downstream gene expression of molecules involved in synaptic maintenance. One possible change is an increase in production of BDNF, which can stabilize synapses through retrograde signaling as we [2] and others [8] have suggested before. However, unless synapses containing VGLUT2 and GAD67 have quite different requirements for or access to BDNF, this self-amplifying mechanism would not explain the selective synaptic withdrawal that we have observed in trkB-KO motoneurons. Alternatively, neurotrophin-trkB binding in motoneurons could lead to an increase in production of other synapse stabilizing molecules whose distribution might be more selective. One interesting class of candidates is adhesion molecules. Expression of adhesion molecules, such as neuroligins and neurexins, has been shown to be essential for normal synapse specification and function in the CNS [37, 38]. Likewise, a reduced expression of the adhesion molecule, Netrin G-2 ligand, has been associated with synaptic withdrawal from axotomized motoneurons [39]. The selective withdrawal of synapses we have observed after elimination of motoneuron trkB receptors might be explained if specific adhesion molecules are regulated by trkB signaling.

Although we show here that signaling through motoneuron trkB receptors is necessary in maintaining some synaptic contacts onto motoneuron somata, the source of the ligand (BDNF and/or NT-4/5) leading to this signaling is unknown. We have assumed an autocrine/paracrine neurotrophin mechanism, where BDNF is secreted by the postsynaptic motoneuron. Current literature supports the function of BDNF as a self-amplifying autocrine factor that elevates cytoplasmic cAMP and protein kinase A activity, which triggers further secretion of BDNF and membrane insertion of trkB [40]. However, we really cannot rule out that BDNF is also released from presynaptic terminals or even surrounding glial cells. Secretion of BDNF at particular presynaptic terminals could act locally to stabilize those synapses. Such a mechanism could be tested by selectively eliminating presynaptic BDNF from afferent neurons containing GAD67 or VGLUT2, or from glial cells, and evaluating synaptic coverage by those types of synaptic structures.

Motoneurons are contacted by synaptic inputs from heterogeneous sources. The maintenance of synaptic contacts from some neurons, such as primary afferent neurons containing VGLUT1 or a subset of interneurons containing only glycine in females, that we observed in the present study clearly does not involve signaling through postsynaptic trkB receptors. This conclusion does not mean that BDNF is not involved in stabilization of these synapses. Indeed knocking out the gene for BDNF in motoneurons resulted in a marked withdrawal of VGLUT1+ synaptic contacts [2]. Instead, we suggest that stabilization of synapses containing VGLUT1 may require a different, more complex cellular mechanism. Such a mechanism would include retrograde signaling from motoneuron BDNF but also might include signaling through the binding of NT-3 to its receptor tropomyosin related kinase C (trkC). Mendell and his colleagues [11] have shown that application of recombinant human NT-3 to the proximal stump of cut nerves resulted in an enormous increase in the amplitude of group IA evoked monosynaptic EPSPs in the axotomized motoneurons, suggesting an influence of NT-3 on VGLUT1+ synaptic inputs. Similarly, application of different neurotrophins to the proximal stumps of cut eye muscle nerves resulted in the restoration of different types of synapses onto motoneuron cell bodies [9, 20].

We observed a significant sex difference in the change in the estimated synaptic coverage by contacts immunoreactive for glycine following trkB knockout, indicating that the maintenance of these synaptic inputs is different in males and females. The reasons that synapse withdrawal was found only in males are not clear. It could be hormonal, a hypothesis that could be tested experimentally. It also could be related to the interaction of the motoneurons with microglia during the process of synapse withdrawal. Such an interaction has long been proposed to explain synaptic withdrawal following peripheral nerve transection [3], and even though such interaction was later questioned [41], the discovery of microglial-derived BDNF actions on neurons in the spinal cord dorsal horn following peripheral nerve injury in male and androgenized female mice [42, 43] may spark renewed interest in the role of microglia on synaptic withdrawal [44]. We do not know if microglia are involved in the withdrawal of glycinergic inputs from motoneurons following knockout of the trkB gene, or how they might be regulated differently during the withdrawal of VGLUT2 and GABAergic terminals but their association and androgens make them an interesting target for future research.

## 5. Conclusions

Withdrawal of some, but not all types of contacts made by structures immunoreactive to synapse-associated antigens in motoneuron-specific trkB knockout mice, suggests the involvement of postsynaptic trkB activation in the maintenance of these inputs. The lack of withdrawal of VGLUT1 immunoreactive contacts and glycinergic contacts in females indicates that their mechanism of stabilization involves a cellular mechanism that does not include postsynaptic trkB activation.

## Figures and Tables

**Table 1:** Antibodies used.

	Antibody	Concentration	Source
Primary	Rabbit polyclonal anti-VGLUT1	1 : 2000	Synaptic Systems
	Rabbit polyclonal anti-VGLUT2	1 : 200	Synaptic Systems
	Mouse monoclonal anti-GAD67	1 : 200	Millipore
	Rabbit polyclonal anti-VGAT	1 : 200	Synaptic Systems
	Mouse monoclonal anti-NeuN	1 : 200	Millipore
	Rabbit polyclonal anti-NeuN	1 : 200	Millipore

Secondary	Goat anti-rabbit Alexa Fluor 647	1 : 200	Invitrogen
	Goat anti-mouse Alexa Fluor 647	1 : 200	Invitrogen
	Goat anti-rabbit Alexa Fluor 546	1 : 200	Invitrogen
	Goat anti-mouse Alexa Fluor 546	1 : 200	Invitrogen
